# The C-type Lectin Receptor-Driven, Th17 Cell-Mediated Severe Pathology in Schistosomiasis: Not All Immune Responses to Helminth Parasites Are Th2 Dominated

**DOI:** 10.3389/fimmu.2019.00026

**Published:** 2019-01-30

**Authors:** Parisa Kalantari, Stephen C. Bunnell, Miguel J. Stadecker

**Affiliations:** Department of Immunology, Tufts University School of Medicine, Boston, MA, United States

**Keywords:** C-type lectin receptor, CD209a, DC-SIGN, *Schistosoma mansoni*, Th17 cells, immunopathology

## Abstract

Schistosomiasis is a major helminthic disease in which damage to the affected organs is orchestrated by a pathogenic host CD4 T helper (Th) cell-mediated immune response against parasite eggs. In the case of the species *Schistosoma mansoni*, the resulting granulomatous inflammation and fibrosis takes place in the liver and intestines. The magnitude of disease varies greatly from individual to individual but in a minority of patients, there is severe disease and death. *S. mansoni* infection in a murine model similarly results in marked strain variation of immunopathology. In the most commonly examined mouse strain, C57BL/6 (BL/6), there is relatively mild hepatic pathology arising in a Th2-dominated cytokine environment. In contrast, CBA mice develop decisively more severe lesions largely driven by proinflammatory IL-17-producing Th17 cells. Dendritic cells (DCs) from CBA mice differ sharply with those from BL/6 mice in that they vastly over-express the C-type lectin receptor (CLR) CD209a (SIGNR5), a homolog of human DC-SIGN, which senses glycans such as those produced by schistosome eggs. Silencing of CD209a, and recent studies with CD209a KO CBA mice have shown that this receptor is crucial to induce the pathogenic Th17 cell response; indeed, CD209a KO mice display markedly reduced immunopathology akin to that seen in BL/6 mice. Mechanistically, CD209a synergizes with the related CLRs Dectin-2 and Mincle to stimulate increased DC production of IL-1β and IL-23, necessary for pathogenic Th17 cell development. These findings denote key molecular underpinnings of disease variability based on selection and function of contrasting Th cell subsets.

## Schistosome Helminths, Human Schistosomiasis, and the Experimental Murine Model

Schistosomes represent a genus of trematode helminths (blood flukes) that have parasitized mammals for millions of years before parasite and host acquired present-day configurations. Infection in humans occurs when free swimming parasite larvae (cercariae), released from fresh water vector snails, penetrate the skin. Over several weeks, the parasites evolve through several stages to finally home in specific venous beds, where adults mate and lay eggs of which a fraction is trapped in regional organs. Infection with the species *Schistosoma mansoni (S. mansoni)* targets the liver and intestines, where the eggs trigger granulomatous inflammation and fibrosis. There is great variation in the severity of the resulting hepatic immunopathology. Most often there is relatively limited symptomatology and good survival; however, a minority of patients develop severe disease leading to portal hypertension, splenomegaly, ascites, gastro-intestinal hemorrhage, and death (1).

Today, schistosomiasis is considered to be the second most important human parasitic disease and remains a major health and socio-economic concern in many parts of the developing world (2). Despite important measures in sanitation, vector control and treatment programs, new cases are on the rise. Expectations to produce a protective vaccine against the parasite have so far not materialized and the specter of parasite resistance to drug therapy looms large. Although substantial progress has been made in elucidating the immune nature of the schistosome egg-induced inflammation, there is still much to be learned about the mechanisms underlying the marked variation in severity of disease, which is the main focus of the present article.

Our laboratory has investigated the immune response and immunopathology in murine infection with *S. mansoni* using a well-established experimental model that bears remarkable resemblance to the human condition, in terms of immunopathogenesis, anatomic localization, and heterogeneity of disease severity among various mouse strains.

## The Spectrum of Host Immune Response and Immunopathology Elicited by Schistosome Eggs

All stages of the schistosome life cycle inside the mammalian host elicit immune responses, but only the eggs are the target of a vigorous granulomatous inflammatory reaction that is the cause of the observed morbidity. The hepatic inflammation is mediated by CD4 Th cells specific for egg antigens, making schistosomiasis an immunologically mediated disease, since the damage to the affected tissues is largely inflicted by the host's own immune system rather than by the parasite itself. However, despite identical infection protocols, there is considerable lesional variation among different inbred mouse strains (3). CBA, C3H, SJL, and MOLF mice all develop large egg granulomas with inflammatory cell spillage into the surrounding hepatic parenchyma, whereas in BL/6 mice, the most commonly used strain in experimental schistosomiasis research, they are more confined and significantly smaller in size (4–7). Additionally, the high-pathology strains display significantly enhanced hepatomegaly, splenomegaly as well as mesenteric lymphadenomegaly. Crosses between the high-pathology strains with BL/6 yield low-pathology F1 offspring, and in F2 cohorts the pathology typically ranges from low to severe (6). Different high- x low-pathology crosses reveal considerable variation in the number and location of the quantitative trait loci involved in the control of immunopathology, indicating that the molecular mechanisms mediating these responses are complex (6). In our studies, we have largely focused on CBA and BL/6 mice as our model high- vs. low-pathology strains.

Cytokines produced by egg antigen-stimulated liver, mesenteric lymph node or spleen T cell populations from infected mice are faithful correlates of immunopathology. In BL/6 mice, an initial short-lived elevation in IFN-γ-producing Th1 cells gives way to a largely host-protective Th2 (IL-4, IL-5, IL-13)-dominated environment (8), whereas high-pathology strains sustain a proinflammatory Th1 and Th17 (IL-17) cell-polarized response alongside the Th2 response (6). The Th1/Th17 phenotype is largely driven by antigen presenting cells (APCs) expressing markers consistent with classical activation, while the Th2 response is supported by alternatively activated APCs (9). A prompt and effective down-modulation of the proinflammatory response is critical to limit immunopathology and enhance host survival (10).

*In vitro* lymphoid cell cultures have traditionally been stimulated with a soluble preparation of schistosome egg antigens (SEA) (11). However, in our lab we have gradually replaced SEA with freshly harvested live eggs, as they obviate the risk of losing temperature-sensitive, short-lived or short-ranged components, and more physiologically embody those released during the *in vivo* infection. A vast array of egg-secreted products, mostly glycoproteins, have been identified (12), with some of the most abundant ones having been the subject of closer analysis. IPSE (SmEP-25) (13), Omega 1 (14), Kappa 5 (15), largely tested in BL/6 mice, stimulate Th2 cytokine production, whereas Sm-p40 elicits a prominent oligoclonal Th1/Th17 cell response in the CBA strain (4, 6, 16, 17).

## Th17 Cells are Drivers of Severe Pathology in Schistosomiasis

In the early 2000's the newly discovered Th17 cells, rather than Th1 cells, were found to play a central role in mediating the immunopathology in a number of autoimmune conditions, including experimental allergic encephalomyelitis, collagen-induced arthritis, psoriasis and colitis (18). Th17 cells are a distinct lineage of proinflammatory CD4 T cells characterized by expressing the master lineage-specific transcription factor RORγt and secreting the key signature cytokine IL-17A, herein termed IL-17 (19, 20). Analogous to the autoimmune diseases, we demonstrated that Th17 cells, rather than the originally presumed Th1 cells, are the main drivers of pathology in schistosome-infected CBA mice (21). Th17 cells were also shown to drive high pathology in a surrogate model afforded by simultaneously immunizing infected BL/6 mice with SEA emulsified in complete Freund's adjuvant (CFA) (9, 22). Neither SEA or CFA by themselves were able to achieve disease exacerbation and a Th17 cell response, suggesting that this effect was due to concurrent stimulation of relevant innate receptors with egg antigens plus mycobacterial antigens contained in CFA (23), analogous to what has also been described with the use of other microbial products (24). Since the description of their role in the development of severe immunopathology in *S. mansoni* infection, Th17 cells were also reported to mediate hepatic egg-induced pathology in murine infection with *S. japonicum* (25–27), and to be associated in humans with bladder pathology caused by eggs of *S. hematobium* (28). Th17 responses have also been associated with *Ascaris suum* infection, as has morbidity in filariasis (29–32).

Among the splenic APC populations purified from egg-stimulated CBA mice, DCs optimally activated Th17 cell responses in *in vitro* co-cultures with T cells, while macrophages, B cells and neutrophils were ineffective in this regard (33). GM-CSF-elicited bone marrow-derived (BMDCs) from CBA mice were also effective inducers of IL-17, whereas BL/6-derived BMDCs induced little to no IL-17 production (33). Analysis of supernatants from live egg-stimulated CBA BMDCs revealed that IL-1β and IL-23 were the critical cytokines inducing Th17 cell differentiation and that they cooperate to achieve maximal IL-17 production by T cells (6, 21, 34).

The secretion of mature IL-1β requires the transcriptional activation of the IL-1β locus and the proteolytic processing of cytoplasmic pro-IL-1β. The latter event is controlled by a family of intracellular immune receptors that are assembled into inflammasomes, which facilitate the activation of caspase-1, enable the cleavage of pro-IL-1β, and trigger the release of bioactive IL-1β (35, 36). A previous relevant study described the participation of the NLRP3 inflammasome in the secretion of IL-1β following stimulation with SEA and a TLR agonist, with neither stimulus being capable of doing so independently (37). This contrasts with our model in which live eggs can deliver all of the signals necessary for IL-1β production by DCs, although the precise identity of these compounds is currently unknown.

## CD209a Expression on BMDCs Enhances the Production of Pathogenic Th17-Associated Proinflammatory cytokines

A genome-wide gene profiling analysis to account for genetic differences between CBA and BL/6 BMDCs surprisingly disclosed a significant disparity in the expression of several C-type lectin receptors (CLRs). CLRs are a family of calcium-dependent pattern recognition receptors bearing conserved carbohydrate-recognition domains that bind a wide variety of host- and pathogen-derived glycans (38). Specifically, expression of CD209a (SIGNR5, mDC-SIGN), a murine homolog of human DC-specific ICAM-3 grabbing non-integrin (DC-SIGN/CD209), was strikingly higher (~18x) in unstimulated BMDCs from CBA mice relative to BL/6 mice (33). Similar changes in CD209a expression were observed in the spleens and liver egg granulomas of infected CBA vs. BL/6 mice by mRNA analysis, flow cytometry, and immunofluorescent tissue staining (33).

To test whether the enhanced expression of CD209a on BMDCs from CBA mice plays a crucial role in the development of egg-induced Th17 responses and severe immunopathology we used lentivirally-expressed short hairpin RNAs (shRNAs) to suppress CD209a in CBA BMDCs. This treatment markedly decreased the production of IL-1β and IL-23 following stimulation with live eggs, and profoundly attenuated IL-17 production by co-cultured CD4 T cells. Conversely, the lentiviral over-expression of CD209a conferred on egg-stimulated BL/6 BMDCs the ability to produce IL-1β and IL-23, and to induce Th17 cells (33, 39). Thus, the relative inability of BL/6 mice to mount pathogenic Th17 responses in response to schistosome eggs cannot be attributed to intrinsic defects present in BL/6 T cells, and is instead linked to the inability of BL/6 BMDCs to elicit these responses.

To facilitate analogous studies *in vivo*, we produced CD209a KO mice backcrossed to the CBA background. After a 7-week infection, these mice were far less prone to hepatic granulomatous inflammation, splenomegaly and mesenteric lymphadenomegaly than their wild-type CBA counterparts, and instead resembled wild-type BL/6 mice. Immune cells from the spleen and livers of the CD209a-deficient CBA mice, as opposed to wild-type CBA mice, produced less IL-1β, IL-23, IL-17, IFNγ, IL-6, and TNFα, and more of the Th2-associated cytokines IL-4, IL-5, and IL-13. The egg-specific recall responses of CD209a KO spleen, MLN and liver granuloma cells yielded similar CD209a-dependent shifts in cytokine production. *In vitro*, CD209a KO CBA BMDCs failed to produce significant IL-1β and IL-23 in response to live eggs, and could not induce effective IL-17 production by CD4 T cells, even though CD209a did not affect the levels of TNF-α. Thus, both *in vivo* and *in vitro*, CD209a selectively enhances proinflammatory pathways associated with the induction of Th17 responses (40).

## The CD209/DC-SIGN Family of CLRs and Their Egg Ligands

Although the functions of CD209a have not been well characterized, human DC-SIGN has been intensively studied given its roles in adhesion, pathogen uptake, and pathogen-dependent signaling. Like most members of this family of CLRs, DC-SIGN is a type II transmembrane protein, with a cytoplasmic tail, a transmembrane region, a tetrameric neck domain, and an extracellular C-type lectin domain. DC-SIGN, present on monocyte-derived DCs and immature tissue DCs, recognizes and internalizes a wide range of mannose and fucose-containing glycans derived from fungi, bacteria, viruses, parasites, and, of relevance to this work, schistosomes (41–43). In addition, DC-SIGN shapes the induction of adaptive cellular and humoral immune responses, favoring Th1 responses when presented with mannosylated ligands, and favoring Th2 or follicular T helper cell (T_FH_) responses when presented with fucosylated ligands (44–46). DC-SIGNR (CD209L), a closely related human paralog, interacts with similar pathogens, but is primarily found on endothelial cells and macrophages and favors mannosylated glycans (41, 47, 48).

The murine receptors within this family are less well understood, as they are not direct orthologs of the human receptors. Of seven paralogous CD209-related proteins, only three clearly function as membrane-bound receptors: CD209a (SIGNR5), CD209b (SIGNR1), and CD209d (SIGNR3) (41, 49). CD209a and CD209d are expressed in DCs, and may function in a manner analogous to DC-SIGN, while CD209b is expressed in a pattern resembling that of DC-SIGNR. The murine CD209 proteins also bind a spectrum of mannose- and fucose-containing ligands (50).

Schistosome eggs express the relevant mannosylated and fucosylated glycans, which are largely attached to glycoproteins. Typical motifs include Lewis X (Le^x^), Poly Le^x^, pseudo Lewis Y (Le^y^), LacdiNac (LDN), and variously fucosylated (F) forms of LDN (F-LDN, LDN-F, F-LDN-F) (51–54). Many of these glycan moieties bind human DC-SIGN, suggesting that murine homologs of DC-SIGN function as receptors for schistosome egg glycans [reviewed in (54)]. Indeed, CD209b interacts with multiple *S. mansoni* antigens *in vitro*; however, the loss of CD209b has no impact on schistosome-induced immunopathology (55).

**Figure 1:**
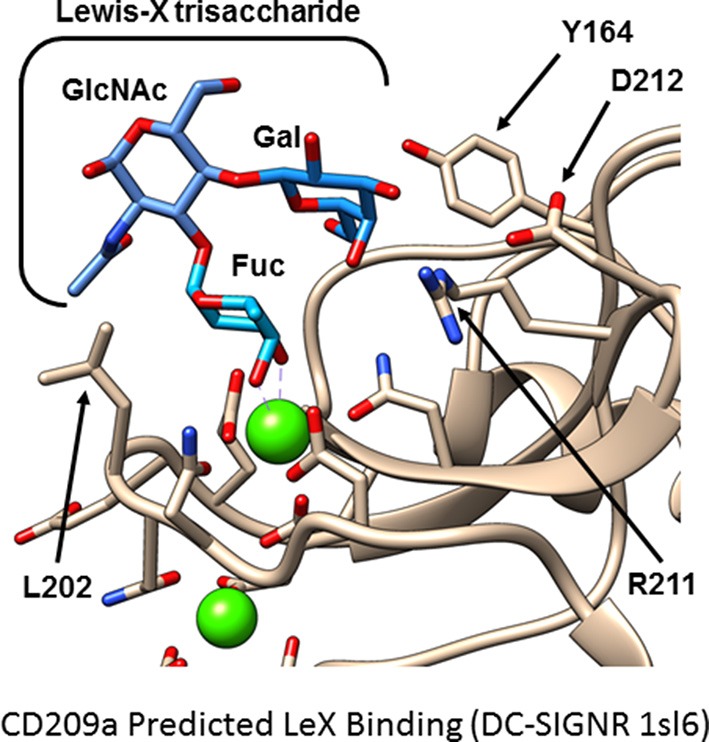
Hypothetical model of CD209a binding Lewis X. A model of murine CD209a was based on an existing DC-SIGNR structure (PDB 1sl6) using the Phyre2 web interface. The Lewis X trisaccharide (blue) is predicted to bind CD209a (tan). Side chains involved in calcium or sugar binding are shown. Calcium ions are shown in green; side chain oxygen and nitrogen atoms are shown in red and blue, respectively. Residues analogous to Y164 and L202 influence ligand selectivity in DC-SIGN and DC-SIGNR. In this model of CD209a, R211, and D212 are oriented toward the ligand-binding site.

Because no ligands of CD209a have been discovered, we examined whether the sugar-binding site in the C-type lectin domain is required for the biological activity of CD209a. Mutations affecting the calcium-dependent fucose- or mannose-binding site prevented the expression of CD209a. However, nearby surfaces involving residues analogous to Y164 and L202 of CD209a influence the specificity of CLR-glycan interactions (56, 57). When we replaced two nearby and distinctive surface-exposed residues with residues common to DC-SIGN, DC-SIGNR, and CD209d (CD209a R211S/D212G, Figure 1) the resulting receptor was surface-expressed but could not enhance the production of proinflammatory cytokines by BL/6-derived BMDCs challenged with live schistosome eggs. This strongly suggests that the specific recognition of a schistosome-derived ligand by CD209a is required to initiate proinflammatory signaling pathways (40).

To clarify the nature of the schistosome-derived ligand(s) that triggers CD209a-dependent signals we generated recombinant CD209a chimeras via a tandem affinity purification tag placed at the amino-terminus of the full-length receptor. Using this recombinant protein, we were able to screen a glycan library for the first time. Although no overwhelming candidates were identified, there was a trend toward the recognition of multi-antennary glycan structures containing Lewis-related antigens, consistent with the properties of the DC-SIGN receptor family.

## CD209a Activates Raf-1 to Promote Inflammation

**Figure 2:**
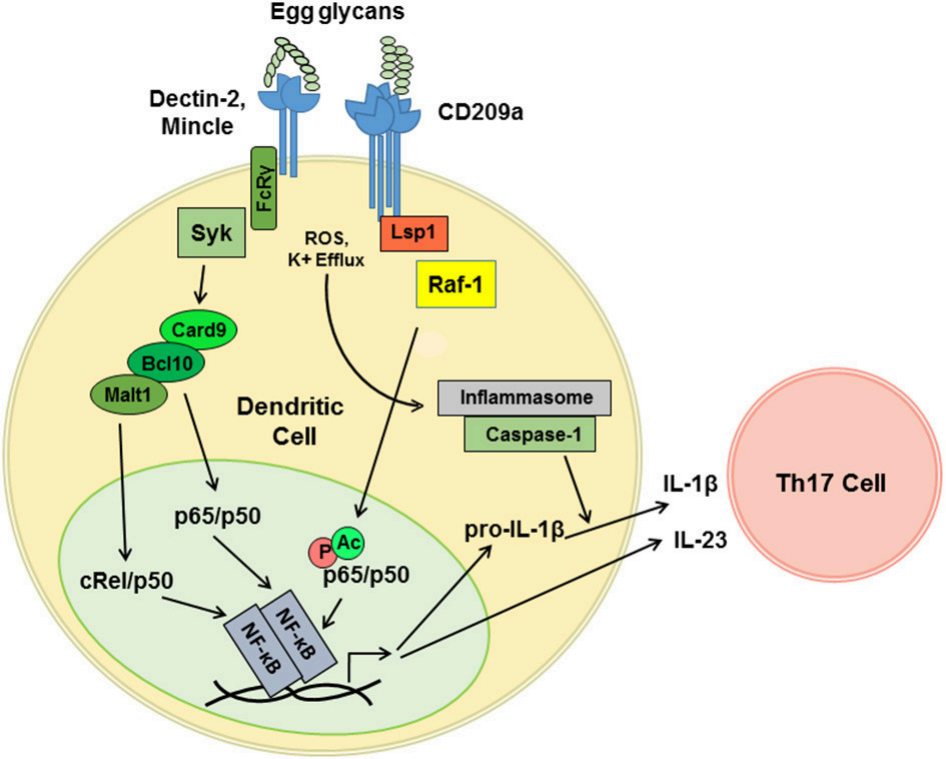
CD209a synergizes with Dectin-2-related receptors to drive the pro-inflammatory cytokine responses triggered by live schistosome eggs. Schistosome egg ligands interact with Dectin-2, Mincle, and CD209a. Dectin-2 and Mincle initiate immunoreceptor-like signals that are mediated by the FcRγ subunit and the tyrosine kinase Syk. Downstream of Syk, Card9 nucleates complexes containing Bcl10 and Malt1, which drive the translocation of the canonical NF-κB heterodimers required for the transcription of proinflammatory cytokine mRNAs. CD209a ligation activates Raf-1 most likely via the scaffold LSP1. By analogy with human DC-SIGN, Raf-1 is predicted to drive the phosphorylation (P) and acetylation (Ac) of p65, and to thereby enhance and sustain the activation of NF-κB. Schistosome eggs also activate the NLRP3 inflammasome, likely via ROS and K^+^ efflux, which enables the processing of pro-IL-1β and secretion of mature IL-1β. IL-1β together with IL-23 induce the differentiation and activation of Th17 cells.

DC-SIGN and CD209a are capable of enhancing the production of proinflammatory Th1- and Th17-associated cytokines following pathogen challenge. The relevant mechanisms are best understood for human DC-SIGN, which signals via a constitutively associated adaptor, lymphocyte-specific protein 1 (LSP1). LSP1 provides a platform for the recruitment of a tripartite “signalosome” consisting of KSR1, CNK, and Raf-1. When mannosylated ligands are delivered concurrently with LPS, this LSP1-associated “signalosome” mediates the activation of Ras and Raf-1. In turn, Raf-1 phosphorylates the canonical NF-κB subunit p65 (RelA) on S276. This directs the acetylation of p65, which enhances the activity of p65 and enables p65 to antagonize non-canonical RelB signaling (44, 58). Although less is known about the mechanisms by which the murine CD209 family members signal, we determined that CD209a-dependent increases in the production of IL-1β and IL-23 following stimulation with live eggs require the activity of Raf-1 (40) (Figure 2). This effect is specific, as perturbations of Raf-1 did not block the production of egg-induced TNFα. Thus, the schistosome egg-induced responses mediated by CD209a may require LSP1-dependent pathways analogous to those employed by human DC-SIGN during the recognition of mannosylated ligands. Although CD209a enhances Th17 responses and promotes immunopathology in murine schistosomiasis, DC-SIGN can also favor the Th2 responses when challenged by fucosylated ligands. To date, the relevant mechanisms have only been studied in human cells, where fucosylated ligands drive the dissociation of the KSR1/CNK/Raf-1 signalosome from LSP1, precluding the activation of Raf-1 (44). Instead, an alternative LSP1-dependent signaling complex is assembled, resulting in the nuclear translocation of Bcl-3, which pairs with p50/p50 homodimers to drive the transcription of Th2-associated cytokines and chemokines (45). It remains to be seen whether the murine homologs of DC-SIGN are capable of directing Th2-promoting events when presented with fucosylated ligands, or whether the murine CD209 family members have evolved fixed, specialized, proinflammatory functions.

## CD209a Drives Inflammation in Synergy With Dectin-2 and Mincle

Our recent work suggests that variable function of DC-SIGN may not need to be dictated by the recognition of distinct ligands but may instead be determined by the combinatorial detection of ligands by additional receptors. In particular, we confirmed that the pattern-recognition receptor Dectin-2 plays a role in the detection of schistosome-derived glycans and revealed that synergy between CD209a and Dectin-2 maximizes the proinflammatory cytokine responses initiated by live schistosome eggs (37, 40). Dectin-2 is a member of a group of CLRs that includes Mincle, Dectin-3, and the dendritic cell activating receptors, Dcar1 and Dcar2 (59). These receptors respond to “non-self” glycans and signal via the gamma subunit of the high affinity IgE receptor (Fcer1g, known as FcRγ), which enables antigen receptor-like signaling via the tyrosine kinase Syk (60, 61). Using egg-challenged BL/6-derived knockout BMDCs expressing CD209a, we determined that Mincle, FcRγ, and Syk also contribute to the induction of proinflammatory signals (40).

In agreement with prior reports revealing a role for the Card9/Bcl10/Malt1 complex downstream of Dectin-2 (62), we demonstrated that the loss of Card9 or the inhibition of Malt1 hinder the production of IL-1β and IL-23 by DCs stimulated with live eggs (40). This is consistent with the role of Malt1 in the Dectin-2-mediated activation of the NF-κB subunit c-Rel, which is a key player in the induction of Th17 responses (63). Therefore, we postulate that a LSP1-dependent, Raf-1-activating pathway initiated by CD209a synergizes with a Card9- and Malt1-dependent pathway initiated by Dectin-2, and, further, that this synergy is mediated via the prolongation of canonical NF-κB signaling and the suppression of the non-canonical and Bcl-3-dependent NF-κB complexes associated with Th2-biased immune responses (Figure 2).

## Conclusions

Infection with schistosomes can result in clinical syndromes of dissimilar severity. In the experimental model, with genetically inbred mouse strains, identical parasite loads and similar infection protocols, disease heterogeneity is largely based on the predominant activation of pro- vs. anti-inflammatory Th cell subsets. Here we have described the synergistic crosstalk of egg antigen-sensing CLRs CD209a with Dectin-2 and Mincle, and analyzed how the interaction of their respective signaling pathways results in the activation of pathogenic Th17 cell responses and the consequent development of severe egg-induced immunopathology. The broader implication of our findings is the knowledge that receptors can collaborate with one another to shape immunopathological responses in a combinatorial fashion and that their signaling pathways can be individually targeted for the purpose of ameliorating disease.

## Author Contributions

All authors listed have made a substantial, direct and intellectual contribution to the work, and approved it for publication.

### Conflict of Interest Statement

The authors declare that the research was conducted in the absence of any commercial or financial relationships that could be construed as a potential conflict of interest.
